# Modulation of RhoA GTPase Activity Sensitizes Human Cervix Carcinoma Cells to *γ*-Radiation by Attenuating DNA Repair Pathways

**DOI:** 10.1155/2016/6012642

**Published:** 2015-11-15

**Authors:** Juliana H. Osaki, Gisele Espinha, Yuli T. Magalhaes, Fabio L. Forti

**Affiliations:** Laboratory of Signaling in Biomolecular Systems, Department of Biochemistry, Institute of Chemistry, University of São Paulo, 05508-000 São Paulo, SP, Brazil

## Abstract

Radiotherapy with *γ*-radiation is widely used in cancer treatment to induce DNA damage reducing cell proliferation and to kill tumor cells. Although RhoA GTPase overexpression/hyperactivation is observed in many malignancies, the effect of RhoA activity modulation on cancer radiosensitivity has not been previously investigated. Here, we generated stable HeLa cell clones expressing either the dominant negative RhoA-N19 or the constitutively active RhoA-V14 and compared the responses of these cell lines with those of parental HeLa cells, after treatment with low doses of *γ*-radiation. HeLa-RhoA-N19 and HeLa-RhoA-V14 clones displayed reduced proliferation and survival compared to parental cells after radiation and became arrested at cell cycle stages correlated with increased cellular senescence and apoptosis. Also, Chk1/Chk2 and histone H2A phosphorylation data, as well as comet assays, suggest that the levels of DNA damage and DNA repair activation and efficiency in HeLa cell lines are correlated with active RhoA. In agreement with these results, RhoA inhibition by C3 toxin expression drastically affected homologous recombination (HR) and nonhomologous end joining (NHEJ). These data suggest that modulation of RhoA GTPase activity impairs DNA damage repair, increasing HeLa cell radiosensitivity.

## 1. Introduction

Radiotherapy is widely used in the clinic to inhibit cancer progression and can be administered as a monotherapy or combined with chemotherapy, surgery, and other alternatives. During *γ*-radiation radiotherapy, the ionizing radiation applied to tumors is absorbed directly by DNA, inducing DNA damage (including single- and double-strand breaks) [1], which leads to tumor cell death or decreases the effect of tumor cells on adjacent tissues.

In the human cervical carcinoma cell line HeLa, treatment with the bacterial toxin HdCDT induces DNA double-strand breaks similar to those resulting from *γ*-radiation [2]. In this system, induction of DNA double-strand breaks activates the small GTPase RhoA, which regulates a variety of cellular activities involving cytoskeletal reorganization (including cell motility and actin stress fiber formation), as well as cell cycle progression [3]. RhoA overexpression has been reported in breast, colon, lung, and gastric tumors, and it facilitates cancer progression by inducing increased tumor cell motility, proliferation, and survival, as well as a loss of cell polarity [4]. Rho family enzymes interchange between an active form (Rho-GTP) and an inactive form (Rho-GDP). GTPase activation by GTP binding is aided by guanine nucleotide exchange factors (GEFs), which catalyze the exchange of GDP by GTP in the active site. The intrinsic activity of GTP hydrolysis of Rho enzymes (including RhoA) is then activated by GAPs (GTPase activating proteins), leading to conversion of GTP into GDP and Rho inactivation [5].

In HeLa cells, RhoA activation by HdCDT treatment increases HeLa cell survival, and this effect depends on the activity of the ataxia telangiectasia mutated (ATM) serine/threonine protein kinase [2], a DNA damage repair protein activated as a response to DNA double-strand breaks (such as those induced by ionizing radiation) [6]. Although these data suggest the existence of a “cross talk” between RhoA and DNA repair pathways, the effect of RhoA activity modulation on the sensitivity of cancer cells to radiotherapy has not been examined to date.

In this study, we analyzed the effect of modulation of RhoA activity in the response to *γ*-radiation (0.5, 5, and 15 Gy) treatment, in HeLa cells. We generated stable HeLa cell lines that express a constitutively active RhoA (HeLa-RhoA-V14) or a dominant negative RhoA (HeLa-RhoA-N19). These mutants are analogous to the Ras-V12 (constitutively active) and Ras-N17 (dominant negative) mutants found in ~25% of all human cancers, in marked contrast to RhoA mutations, which are rarely found [7, 8]. Our results show that cells expressing either the constitutively active or the dominant negative RhoA mutants are less resistant to the effects of *γ*-radiation than parental HeLa cells and have reduced ability to proliferate and survive after treatment. These data correlated with the reduced activation of DNA damage response and repair pathways and efficiency of DNA damage repair, in cells with reduced RhoA activity.

## 2. Materials and Methods

### 2.1. Cell Lines

The human cervical carcinoma cell line HeLa (CCL-2; ATCC, Manassas, VA, USA) was maintained in Dulbecco's Modified Eagle Medium (DMEM; Invitrogen, Waltham, MA, USA) supplemented with 10% fetal bovine serum (FBS; Cultilab, Campinas, SP, Brazil), at 37°C and 5% CO_2_, in a humidified incubator.

### 2.2. Cell Treatments by *γ*-Radiation

HeLa cells and clones were treated with three different doses (0.5, 2, 5, and 15 Gy) of gamma (*γ*) ionizing radiation (Co60-Gammacell 220, Atomic Energy of Canada Limited (AECL), Ontario, Canada) at the Nuclear and Energy Research Institute (IPEN, SP, Brazil). But for some experiments dose-response curves were performed, while for others only one dose was used, according to the cell viability (unpublished results, not shown) and the duration of the experiment.

### 2.3. Generation of Sublines of RhoA-N19 and RhoA-V14 Mutants from HeLa Cells

To produce HeLa cell sublines stably expressing RhoA mutants, constructs containing the recombinant retroviral pCM vector and cDNA sequences for the constitutively active RhoA-V14 (Ala to Val substitution at position 14) or the dominant negative RhoA-N19 (Thr to Asp substitution at position 19) were packaged into recombinant retrovirus particles using the Phoenix system (*ϕ*NX-cells, kindly donated by Gary P. Nolan, Stanford University, CA, USA). Subconfluent HeLa cells seeded in 10 cm dishes (in DMEM/10% FBS) were infected with the recombinant retrovirus particles in the presence of 8 *μ*g/mL of polybrene [9]. After infection, cells were selected for approximately 30 days with 500 *μ*g/mL of G418, and isolated colonies, representing clones of HeLa-RhoA-N19 and HeLa-RhoA-V14, were collected and maintained in DMEM/10% FBS with 100 *μ*g/mL of G418 until freezing or further use.

### 2.4. Active RhoA Pull-Down Assay

To measure RhoA activity, we used a RhoA-GTP pull-down protocol adapted from Ren et al., 1999 [10]. HeLa cells were lysed in RIPA buffer (50 mmol/L Tris-HCl, pH 7.2, containing 1% Triton X-100, 0.5% sodium deoxycholate, 0.1% SDS, 500 mmol/L NaCl, 10 mmol/L MgCl_2_, 1 mM Na_3_Vo_4_, 1 mM NaF, 2 *μ*g/mL leupeptin, pepstatin, aprotinin, and 1 mmol/L phenylmethylsulfonyl fluoride, or PMSF) (all from Sigma-Aldrich, Saint Louis, MO, USA), and cell lysates were incubated with Glutathione-Sepharose beads (GE, Healthcare, Cleveland, OH, USA) bound to the RBD-GST fusion protein (RhoA binding domain of the Rhotekin protein, kindly donated by Gary M. Bokoch, The Scripps Research Institute, La Jolla, CA, USA) for 90 min at 4°C. Then, beads were recovered by centrifugation (3000 rpm, for 3 min at 4°C) and washed 3 times with buffer B (50 mmol/L Tris-HCl, pH 7.2, containing 1% Triton X-100, 150 mmol/L NaCl, 10 mmol/L MgCl_2_, 1 mM Na_3_Vo_4_, 1 mM NaF, 10 *μ*g/mL leupeptin, aprotinin, and 0.1 mmol/L PMSF). RhoA-GTP bound to RBD-GST-Sepharose beads was resolved on 13% SDS-PAGE gels, transferred to nitrocellulose membranes and analyzed using a monoclonal anti-RhoA antibody (26C4, from Santa Cruz Biotechnology, Santa Cruz, CA, USA), as described below (see Section 2.11).

### 2.5. Growth Curves

For population growth analysis, HeLa cells were seeded in 35 mm dishes (3.5 × 10^4^ cells/dish) and allowed to adhere at 37°C (with 5% CO_2_), for 24 h. Then cells were exposed to 0.5 or 5 Gy of *γ*-radiation and reincubated at 37°C. Cell samples were collected in duplicate every 24 h after *γ*-radiation, for five consecutive days, and cells were counted manually in a Fuchs-Rosenthal chamber.

### 2.6. Clonogenic Assays

For clonogenic assays, HeLa cells were seeded in 60-mm dishes (2 × 10^3^ cells/dish) and allowed to adhere at 37°C (with 5% CO_2_) for 24 h. Then, cells were exposed to 0.5, 5, or 15 Gy of *γ*-radiation and reincubated at 37°C for 10–12 days. Colony foci were fixed in 10% formaldehyde in PBS for 10 min, stained with 0.5% crystal violet in PBS for 5 min (both at room temperature), and counted manually.

### 2.7. Cell Cycle Analysis

For cell cycle analysis, HeLa cells were plated in 35-mm dishes (3.5 × 10^5^ cells/dish) and allowed to adhere at 37°C (with 5% CO_2_) for 24 h. After *γ*-radiation, cells were harvested by trypsinization, washed in PBS, and fixed in 80% ethanol in PBS. Then, cells were stained with 10 *μ*g/mL propidium iodide (PI) and stored at 4°C. Samples were run in a Beckman Coulter FC500 MPL cytometer (Brea, CA, USA), and flow cytometry data were analyzed using WinMDI 2.8 software (Purdue University Cytometry Laboratories, West Lafayette, IN, USA).

### 2.8. Apoptosis Assay

To estimate apoptosis, HeLa cells were plated in 35-mm dishes (1.5 × 10^5^ cells/dish), for 24 h, and treated with 5 Gy or 15 Gy of *γ*-radiation, or with 60 J/m^2^ ultraviolet C (UVC; positive control for apoptosis induction). Then, adhered and suspended cells were harvested by successive rounds of PBS washing-trypsinization-centrifugation, 48 h or 72 h after *γ*-radiation. Harvested cells were resuspended in Annexin-V binding buffer (50 mM HEPES, pH 7.4, containing 0.7 M NaCl and 12.5 mM CaCl_2_) for a final density of 1 × 10^6^ cells/mL, and 5 *μ*L Annexin-V-FITC (BD Biosciences, Franklin Lakes, NJ, USA) and 1.5 *μ*L propidium iodide (1 mg/mL) were added to 100-*μ*L aliquots of cell suspension (1 × 10^5^ cells). Samples were incubated for 15 min at room temperature (and protected from light), and then 400 *μ*L of Annexin-V binding buffer was added to each sample, and cells were analyzed by flow cytometry in a FACSVerse (BD Biosciences, Franklin Lakes, NJ, USA). Flow cytometry data were analyzed on the Kaluza 1.3 Flow Analysis software (Beckman Coulter, Brea, CA, USA).

### 2.9. Senescence-Associated *β*-Galactosidase Assay

Cell senescence was estimated using a senescence-associated *β*-galactosidase assay, as described by Dimri et al., 1995 [11]. HeLa cells (3.0 × 10^4^ cells/dish, in 35-mm dishes) (Corning, New York, NY, USA) were allowed to adhere at 37°C (with 5% CO_2_) for 24 h, prior to treatment with 0.5, 5, or 15 Gy of *γ*-radiation. Then, cells were incubated for 96 h at 37°C, fixed in 2% formaldehyde/0.2% glutaraldehyde in PBS for 3 min, washed in PBS, and stained for 18 h at 37°C with 2 mL/dish of X-gal staining solution (30 mmol/L PBS/citric acid (pH 6) containing 5 mmol/L K_3_Fe(CN)_6_, 2 mmol/L MgCl_2_, 150 mmol/L NaCl, 5 mmol/L K_4_Fe(CN)_6_, and 1 mg/mL X-gal). Then, samples were washed twice in PBS and kept at 4°C prior to analysis, by direct counting of *β*-galactosidase-positive/negative cells (1 × 10^3^ cells/dish, in duplicate), in an inverted Olympus microscope (Olympus, Tokyo, Japan).

### 2.10. Alkaline Comet Assay

The alkaline comet assay was performed as described by Singh et al., 1998 [12], with modifications. HeLa cells were seeded in 35-mm dishes (2 × 10^5^ cells/dish) and were allowed to adhere at 37°C (with 5% CO_2_) for 24 h, before *γ*-radiation with 5 Gy. After treatment, cells were harvested by trypsinization, mixed with 0.5% low-melting point agarose, and 100 *μ*L of this mixture was pipetted onto glass slides with 1.5% normal-melting point agarose. Then, cells were lysed with lysis buffer (10 mmol/L Tris, pH 10, containing 2.5 mmol/L NaCl, 100 mmol/L EDTA, 1% Triton X-100, and 10% DMSO, all from Sigma-Aldrich, Saint Louis, MO, USA) for 24 h at 4°C and in the dark. Samples were denatured in alkaline electrophoresis buffer (300 mmol/L NaOH, 1 mmol/L EDTA, pH >13) for 25 min, and then electrophoresis was performed at 25 V and 300 mA, for 30 min. After electrophoresis, slides were washed 3 times (5 min/wash) in neutralizing buffer (0.4 mmol/L Tris-HCl, pH 7.5), DNA was stained with 2 *μ*g/mL ethidium bromide, and comets (from 50 cells/slide, in duplicate) were imaged using a fluorescence microscope Olympus IX51 (Olympus, Shinjuku, Tokyo, Japan). Comet assay data were analyzed using the software Komet 6.0 (Andor, Technology, Belfast, BT, UK).

### 2.11. Western Blotting

For Western blotting, HeLa cells were lysed with RIPA buffer (see Section 2.4), and 50 *μ*g of protein was mixed with Laemmli sample buffer [13] and resolved in 12% SDS-PAGE gels. Proteins were transferred to nitrocellulose membrane (Millipore, Billerica, MA, USA), and membranes were blocked in TBS-T with 5% milk, for 1 h at room temperature. Then, membranes were incubated with one of the following primary antibodies diluted in TBS-T: anti-phospho-Chk1 Ser345 (Cat. number 2341), anti-phospho-Chk2 Thr-68 (Cat. number 2661), or anti-phospho-H2AX Ser139 (Cat. number 9718) polyclonal/monoclonal antibodies from Cell Signaling (Danvers, MA, USA) or an anti-*α*-Tubulin polyclonal/monoclonal antibody (B-7, Santa Cruz Biotechnology, Santa Cruz, CA, USA). Membranes were incubated with appropriate species-specific IRDye (Infrared Dye) secondary antibodies (680 or 800 nm, diluted to 1 : 15000 in TBS-T) for 1 h and visualized and analyzed (by band density quantification) using an Odyssey Infrared Imaging System and the Odyssey V3.0 software (both from Li-COR Biosciences, Lincoln, NE, USA).

### 2.12. Inhibition of RhoA Activity by the C3 Toxin

HeLa cells were transiently transfected with the eukaryotic expression vector pEF-myc (Invitrogen) containing the C3 toxin coding sequence (plasmid kindly provided by Professor Dr. Gary Bokoch, The Scripps Research Institute, La Jolla, CA, USA). HeLa cells were transfected using Lipofectamine 2000 (Invitrogen), according to the manufacturer's instructions, and then plated into 100-mm dishes (for immunoblotting experiments) or 35-mm dishes (for comet assays) and allowed to grow until ~80% confluence. The cells were incubated for 24 hours, prior to RhoA activation analysis (see Section 2.4), and after this time the cells were exposed to 5 Gy of *γ*-radiation and analyzed according to the previously described experiments.

### 2.13. Homologous Recombination (HR) and Nonhomologous End Joining (NHEJ) Assays

The rates of HR and NHEJ were estimated using HeLa cells stably expressing DR-GFP and EJ-GFP, respectively, as described by Gunn and Stark with modifications [14]. To produce HeLa-DR-GFP and HeLa-EJ5-GFP stable cell lines, subconfluent HeLa cells grown in 60-mm dishes were transfected using 7.5 *μ*g Lipofectamine 2000 (Invitrogen, Waltham, MA, USA) and 3.5 *μ*g of DR-GFP or EJ5-GFP plasmids [14], in DMEM with 10% FBS. Transfectants were selected and isolated using 5 *μ*g/mL of puromycin and maintained in DMEM/10% FBS supplemented with 1 *μ*g/mL of puromycin.

For HR and NHEJ assays, approximately 2 × 10^5^ HeLa-DR-GFP and HeLa-EJ5-GFP cells were seeded in 35-mm dishes and allowed to adhere at 37°C (with 5% CO_2_) for 24 h. Then, cells were transfected with 4 *μ*g of the I-*Sce*I expression vector or an empty vector (EV), alone or in combination with 2 *μ*g of pEF-myc-C3 (using 2 *μ*g of Lipofectamine 2000; Invitrogen, Waltham, MA, USA). Cells were harvested 72 h after transfection, and the percentage of GFP-positive cells was determined by flow cytometry in a FACSVerse cytometer (BD Biosciences, Franklin Lakes, NJ, USA).

### 2.14. Statistical Analysis

Comparisons between treatments were performed by Student's *t*-test (for paired data) or by ANOVA (for multiple groups), using the Prism 6.0 software, and differences were considered statistically significant when *P* < 0.05.

## 3. Results

### 3.1. Expression of Dominant Negative or Constitutively Active RhoA Prevents the Increase in Active RhoA Levels by *γ*-Radiation

**Figure 1:**
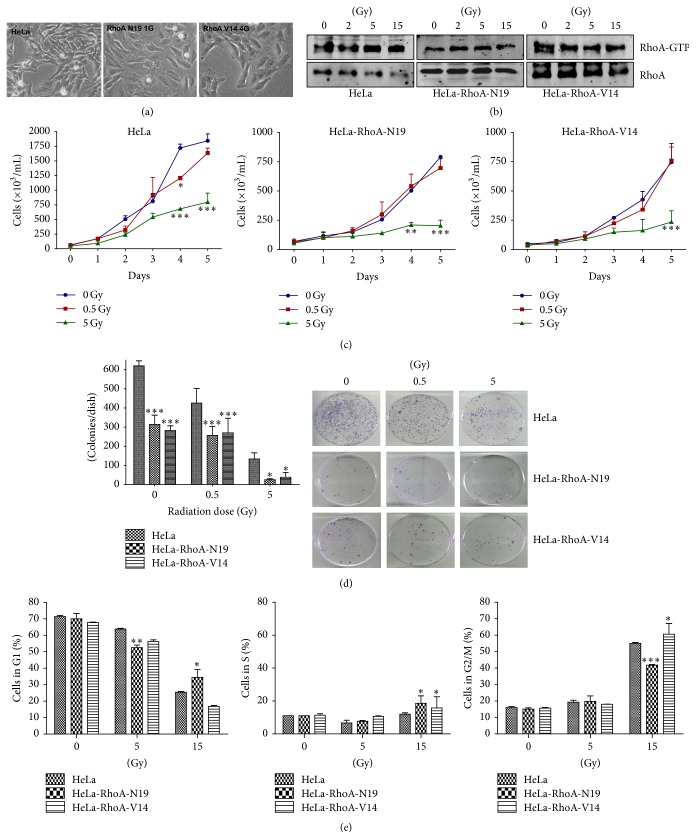
Morphological Rho activity and proliferation analyses of parental and clonal HeLa cell lines expressing RhoA-N19 or RhoA-V14 mutants after *γ*-radiation. (a) Morphology of parental and derived HeLa cell lines. (b) Immunoblotting of pull-down assays for active RhoA (RhoA-GTP) in different cell lines. (c) and (d) Growth curves (c) and clonogenic assays (d) in cell lines under positive or negative modulation of RhoA activity. (e) Cell cycle profiles by flow cytometry analysis (using PI staining) of HeLa cell lines after exposure to different doses of *γ*-radiation. Graphs display mean ± SD of at least three independent experiments. ^*∗*^ *P* < 0.05, ^*∗∗*^ *P* < 0.005, and ^*∗∗∗*^ *P* < 0.001 between clones and parental HeLa cells in the same treatment conditions (by ANOVA).

To evaluate if the activation of the small GTPase RhoA has a role in the response to *γ*-radiation in cancer cells, we generated stable clones of HeLa cells expressing either the constitutively active HeLa-RhoA-V14 or the dominant negative HeLa-RhoA-N19 RhoA mutants. Cells from both clones appeared to spread on the surface of culture flasks more effectively than parental HeLa cells (Figure 1(a)). Analysis of RhoA activity by a pull-down assay for the active RhoA-GTP form [15] showed that HeLa-RhoA-N19 and parental HeLa cells had similar basal levels of active RhoA, while HeLa-RhoA-V14 cells had higher levels of active RhoA, as expected for cells expressing a constitutively active RhoA mutant (Figure 1(b)). RhoA-GTP levels increased after *γ*-radiation in parental HeLa cells (Figure 1(b)). In contrast, we detected no further RhoA activation in cells expressing RhoA-N19 or RhoA-V14, after *γ*-radiation (Figure 1(b)).

The RhoA GTPase is a key regulator of cell migration via cytoskeletal reorganization [16]. Thus, we also performed scratch assays in confluent cell monolayers, to evaluate the effect of mutant RhoA expression on cell migration (see Supplementary Figure S1 in Supplementary Material available online at http://dx.doi.org/10.1155/2016/6012642). For cells grown in medium containing 10% FBS, the migration rate of those expressing the dominant negative RhoA-N19 was considerably reduced (43%) compared with that of parental HeLa or HeLa-RhoA-V14 cells (100% migration). In serum-free conditions (0% FBS), HeLa-RhoA-N19 migrated only 5%, 24 h after serum starvation, but migration rates in 10% FBS (after serum starvation) were similar to those observed in cells that had not been serum-starved prior to migration. However, in starving conditions, HeLa-RhoA-V14 cells displayed reduced migration (27%) compared with parental HeLa cells (Supplementary Figure S1). These results suggest that the expression of either RhoA-V14 or RhoA-N19 promotes an imbalance in the RhoA activity in HeLa cells, despite the presence of normal to high basal levels of active RhoA. While the migration of parental or HeLa-RhoA-V14 cells was only significantly affected by high doses of *γ*-radiation (15 Gy), treatments as low as 5 Gy of *γ*-radiation reduced significantly the migration of HeLa-RhoA-N19 cells in medium with 10% FBS (Supplementary Figure S2). Overall, the scratch assay data suggest that, despite the persistent levels of RhoA-GTP in cells expressing either dominant negative or constitutively active RhoA mutants, these cells had altered RhoA activity, judging from their reduced migration ability, and this effect was particularly evident in cells expressing the dominant negative RhoA-V14 mutant, after *γ*-radiation treatment. Thus, the migration data also indicate that the HeLa-RhoA-V14 and HeLa-RhoA-N19 cell lines are valid models for the study of RhoA activity modulation after radiation.

### 3.2. Expression of RhoA Mutants Alters HeLa Cell Proliferation and Survival Rates after *γ*-Radiation

To investigate the effect of RhoA activity modulation on cell proliferation and survival after exposure to low doses (0.5 and 5 Gy) of *γ*-radiation, we performed growth curves and clonogenic assays of HeLa cells expressing mutant RhoA proteins. HeLa-RhoA-N19 and HeLa-RhoA-V14 displayed a reduced doubling time compared to parental HeLa cells (~3.1 and ~2.6 days, resp.). We observed a clear reduction in the proliferation of all cell lines after exposure to 5 Gy of *γ*-radiation, and proliferation inhibition was observed earlier (between 2 and 3 days after radiation) in HeLa-RhoA-N19 and HeLa-RhoA-V14 cultures, compared with parental HeLa cells (Figure 1(c)). No significant reduction in cell proliferation was observed after exposure to 0.5 Gy of *γ*-radiation (Figure 1(c)).

In clonogenic (colony formation) assays, both HeLa-RhoA-N19 and HeLa-RhoA-V14 displayed decreased survival, with a reduction of ~50% in the number of colonies in untreated cells, and this sensitivity to the effects of 0.5 Gy of *γ*-radiation was relatively well maintained compared with HeLa cells. When exposed to 5 Gy of *γ*-radiation, HeLa-RhoA-V14 and HeLa-RhoA-N19 were significantly more sensitive than parental HeLa cells, with a reduction of ~70% and ~80%, respectively, in the number of colonies, compared with parental cells subjected to the same treatment (Figure 1(d)). These data suggest that HeLa cells expressing either a dominant negative or a constitutively active RhoA mutant are more sensitive to low doses of *γ*-radiation than parental HeLa cells.

### 3.3. Expression of RhoA Mutants Leads to Differential Cell Cycle Arrest with Increased Senescence and Apoptosis Induction

Cell cycle analysis (by flow cytometry using PI) of irradiated cells suggested that, after exposure to 15 Gy of *γ*-radiation, cells with constitutively high levels of activated RhoA (HeLa-RhoA-V14) remain arrested in S and G2/M, whereas HeLa cells expressing the dominant negative RhoA-N19 remain predominantly in the G1 and S phases of the cell cycle (Figure 1(e)). As expected, we observed a marked arrest of HeLa cells in the G2/M phase of the cell cycle after treatment with high (15 Gy) dose of *γ*-radiation (Figure 1(e)).

**Figure 2:**
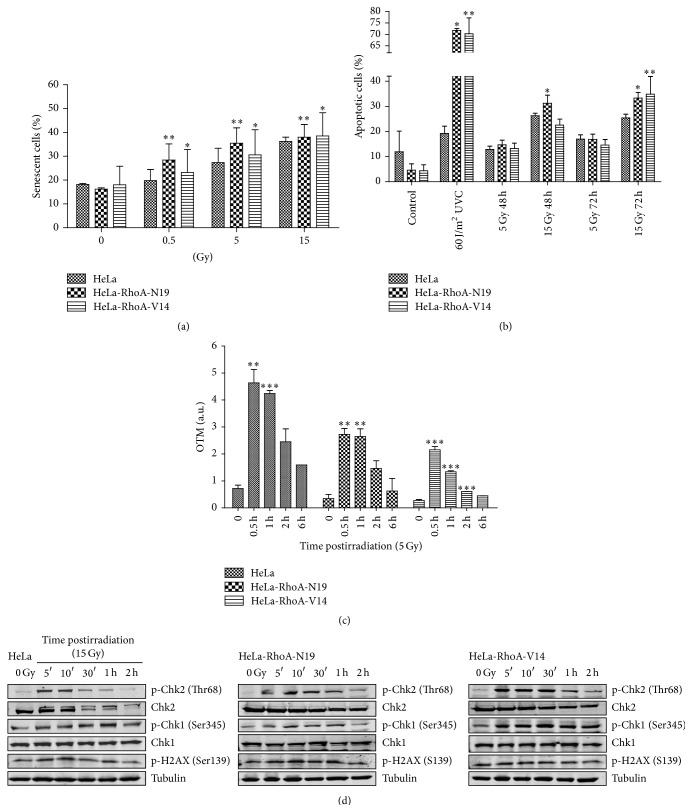
Cell death and DNA damage response and repair analyses in HeLa cells expressing RhoA mutants, after *γ*-radiation treatment. (a) Quantification of senescent cells, by a senescence-associated *β*-galactosidase assay, performed 96 h after radiation. (b) Quantification of apoptotic cell death after radiation, by Annexin-V and propidium iodide staining (Annexin V^+^/PI^+^ cells were considered apoptotic). (c) Estimation of DNA damage and repair efficiency following radiation, by the olive tail moment (OTM, in arbitrary units) measurements from comet assays. (d) Immunoblotting analysis of phosphorylated Chk1/Chk2 and histone H2AX levels in the different HeLa cell lines, after exposure to 15 Gy of *γ*-radiation (and using *α*-Tubulin as loading control). Graphs and immunoblots are representative of three independent experiments. ^*∗*^ *P* < 0.01, ^*∗∗*^ *P* < 0.05, and ^*∗∗∗*^ *P* < 0.001 between clones and parental HeLa cells and ^#^ *P* < 0.005 between treated and untreated conditions (by ANOVA).

The radiation-induced arrest at different cell cycle stages correlates with the distinct types of antiproliferative effects observed in HeLa cell lines expressing RhoA mutants, after radiation treatment (Figure 2). Cells expressing HeLa-RhoA-N19, expected to be deficient in RhoA activity, display higher senescence levels at lower doses of 0.5 and 5 Gy of *γ*-radiation, which correlates with their preferential arrest at G1 and S phases (Figure 2(a)). These cells also showed increased apoptosis 48 and 72 h after exposure to the highest (15 Gy) dose of *γ*-radiation (Figure 2(b)). Similarly, all doses of radiation treatment led to increased senescence in cells expressing the constitutively active HeLa-RhoA-V14 mutant, compared with parental HeLa cells (but not with cells expressing RhoA-N19), although the highest senescence levels were observed only after treatment with the highest dose (15 Gy) of *γ*-radiation (Figure 2(a)). These results correlate with the preferential arrest of HeLa-RhoA-V14 at S and G2/M and with an increase in apoptotic cell death after exposure to 15 Gy of *γ*-radiation, especially at the longer time-point of 72 h posttreatment (Figure 2(b)). Taken together, the cell cycle analysis, apoptosis, and senescence data suggest that modulation of RhoA activity leads to arrest at different stages of the cell cycle, leading to the induction of different levels of senescence or apoptosis.

### 3.4. Modulation of RhoA Activity in HeLa Cells Affects DNA Damage Repair Induction and DNA Damage Response (DDR) Protein Activation

To investigate if modulation of RhoA activity affects DNA repair after radiation treatments, we performed comet assays at different time-points after exposure to 5 Gy of *γ*-radiation. All three HeLa cell lines exhibited a peak of fragmented DNA (i.e., an increase in the olive tail moment, or OTM) 0.5 h after radiation, and this peak was ~6- and ~8-fold higher than the basal levels (in nonirradiated cells), for parental HeLa and RhoA mutant-expressing cells, respectively (Figure 2(c)). Although fragmented DNA levels decreased up to 6 h after radiation in all three HeLa cell lines, DNA repair (i.e., a statistically significant reduction in OTM) could be detected as early as 1 h after radiation treatment in parental HeLa and in HeLa-RhoA-V14 cells (Figure 2(c)). In contrast, DNA repair could be detected from 2 h after *γ*-radiation treatment in HeLa-RhoA-N19 cells, suggesting that DNA damage repair is delayed in this cell line, which is expected to have reduced RhoA activity (Figure 2(c)).

To examine a possible correlation between the efficiency of DNA repair and the phosphorylation of DNA damage responses (DDR) proteins, we exposed parental HeLa, HeLa-RhoA-N19, and HeLa-RhoA-V14 cells to 15 Gy of *γ*-radiation and monitored the activation (by phosphorylation) of the checkpoint proteins Chk1 and Chk2, as well as the appearance of a marker for double-strand DNA breaks (p-Ser139 H2AX), for up to 2 h after radiation (Figure 2(d)). After treatments, we observed a reduction in the phosphorylation levels of both Chk1 (Ser345) and Chk2 (Thr-68) in cells expressing the dominant negative RhoA-N19 mutant, while cells expressing the constitutively active RhoA-V14 displayed overactivation of Chk1/Chk2; both responses were different from those observed in parental HeLa cells. Thus, the levels of Chk1/Chk2 phosphorylation obtained for the three cell lines correlate with their RhoA-GTP levels. In contrast, the phosphorylation of H2AX (Ser139), which peaked between 5 min and 1 h after radiation and returned to basal levels 2 h after treatment, was not significantly affected by the modulation of RhoA activity (Figure 2(d)).

### 3.5. C3 Toxin-Mediated Downregulation of RhoA Activity Impairs DNA Repair and Overactivates DDR Proteins

**Figure 3:**
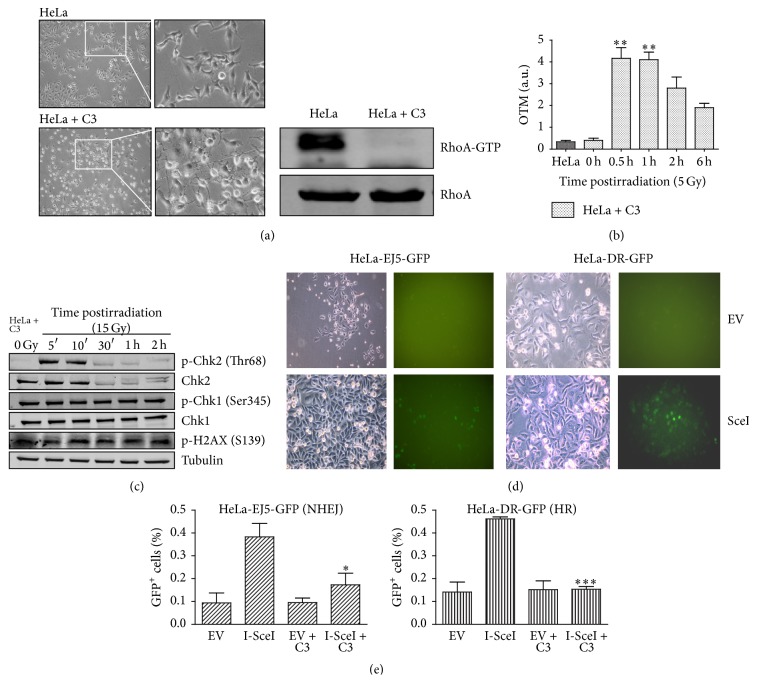
Inhibition of RhoA activity by C3 toxin expression strongly affects DNA damage response, including global and specific DNA repair mechanisms in HeLa cells, following *γ*-radiation. (a) Dendritic morphology of HeLa cells (HeLa + C3 images) associated with decreased RhoA-GTP levels (on the right), 24 h after transfection with a plasmid for C3 toxin expression. Images on the right are insets from those on the left, 200x. (b) Estimates of DNA damage and repair efficiency (by olive tail moment, or OTM, measurements from comet assays) in HeLa cell expressing the C3 toxin, following *γ*-radiation. (c) Immunoblotting analysis of the effects of *γ*-radiation (15 Gy) on phosphorylated Chk1/Chk2 and histone H2AX levels in HeLa cells expressing the C3 toxin (using *α*-Tubulin as a loading control). (d) and (e) Assays for GFP-based detection of homologous recombination (HR, using HeLa-DR-GFP) or nonhomologous end joining (NHEJ, using HeLa-EJ5-GFP) after DNA damage induced by I-*Sce*I restriction enzyme expression. (d) Phase contrast (left) and green fluorescence (right) images of cells transfected with a plasmid for I-*Sce*I expression (I-*Sce*I), or with an empty vector (EV), showing the appearance of GFP-positive cells indicative of HR (HeLa-EJ5-GFP) or NHEJ (HeLa-DR-GFP), 72 h after transfection. (e) Quantification of HR and NHEJ assays, with (EV + C3 and I-*Sce*I + C3 groups) or without (EV and I-*Sce*I groups) concomitant C3 toxin expression. Graphs (with mean ± SD values) and immunoblots are representative of three independent experiments. ^*∗*^ *P* < 0.05, ^*∗∗*^ *P* < 0.001, and ^*∗∗∗*^ *P* < 0.005, between treated and untreated conditions (by ANOVA).

To confirm that decreased RhoA activity reduces DNA repair efficiency, as suggested by the comet assay data on HeLa cells expressing dominant negative RhoA, we performed a potent and persistent inhibition of RhoA in parental HeLa cells by transfection with a plasmid encoding the C3 toxin. To that we transfected HeLa cells with a plasmid driving constitutive expression of the C3 toxin, an exoenzyme secreted by the bacterium* Clostridium botulinum* and capable of selectively inhibiting the activation of RhoA, RhoB, and RhoC GTPases [17–19]. As expected, expression of the C3 toxin had a strong effect on cell morphology, 24 h after transfection, and reduced RhoA-GTP to residual levels (Figure 3(a)). Also, comet assay results suggest that C3 toxin expression increased HeLa cell sensitivity to DNA damage by *γ*-radiation (5 Gy) (Figure 3(b)). After C3 toxin expression, the levels of DNA breaks increased by ~10-fold at 0.5 h after radiation, and DNA damage repair could be detected from 2 h after radiation, similar to the response observed in the HeLa Rho-N19 cells (Figure 2(c)); however, DNA damage appeared more persistent in HeLa cells expressing C3, judging from the levels of damage remaining up to 6 h after *γ*-radiation (Figure 3(b), compared with HeLa, in Figure 2(c)). These results are in agreement with the overactivation of phospho-Chk1 (Ser345) after *γ*-radiation (15 Gy) treatment, in cells expressing the C3 toxin, which seems to reflect the persistence of high phospho-H2AX (Ser139) levels in HeLa cells (Figure 3(c)).

To investigate the effect of C3 toxin-mediated RhoA inhibition on the activity of specific DNA repair pathways, we generated HeLa cell lines capable of GFP-based detection of homologous recombination (HR, via the reporter EJ5-GFP) or nonhomologous end joining (NHEJ, via the reporter gene DR-GFP), after expression of the endonuclease I-*Sce*I, which cleaves on specific sequences in the reporter gene plasmidial DNA [14]. Interestingly, in cells expressing both the C3 toxin and I-*Sce*I, the levels of double-strand break repair by either HR or NHEJ were significantly reduced compared with those observed in cells expressing the I-Sce-I enzyme only, reaching similar levels to those observed in controls (empty vector, or EV, and EV + C3) (Figures 3(d) and 3(e)). Endogenous RhoA inhibition by C3 expression affected both repair pathways: while HR was completely inhibited, NHEJ was partially disrupted in cells where the endogenous repair machinery was specifically recruited to reporter gene sequences (EJ-GFP and DR-GFP, resp.) integrated in the genome. Altogether, these results strongly support the involvement of RhoA in DNA damage response and repair mechanisms.

## 4. Discussion

RhoA GTPase is overexpressed and overactivated in cancer and is involved in cancer progression, directly regulating cell proliferation, survival, and invasion [3, 4]. Our results, using stable HeLa cell lines expressing either a constitutively active RhoA (RhoA-V14) or a dominant negative version of this protein (RhoA-N19), suggest that RhoA GTPase activity also regulates cancer cell sensitivity to *γ*-radiation, by affecting basic DNA repair mechanisms. Despite the fact that HeLa cells have been used as a good model for our hypothesis and this whole work has been done solely on it, we believe that our results do not reflect a cell line-dependent phenomenon because unpublished results (not shown) performed in metastatic melanoma MeWo cell line culminate in similar cellular responses.

We observed that HeLa cells have high basal level of RhoA GTPase in the active state (RhoA-GTP) and that the activity of RhoA was modulated accordingly (up or down) in both mutant clones. RhoA-GTP levels increased in response to either *γ*-radiation activation or serum stimuli (not shown). The high basal levels of RhoA-GTP observed here in cervical adenocarcinoma HeLa cells are similar to those reported for other cancer cell lines, including the breast cancer cell line MDA-MB-231 [20], and also in colorectal cancer cell lines and tumor samples [21]. The RhoA GTPase directly regulates cytoskeletal dynamics via actin polymerization, mediating cell adhesion and migration [16, 22]. In glioblastoma multiforme tumors, radiation-induced activation of RhoA increases cell migration and invasive potential [23]. Our study extends these results, showing that cells expressing the dominant negative RhoA-N19 display decreased migration rates, both in the presence and in the absence of FBS, and also following *γ*-radiation. The opposite was observed for HeLa cells expressing the constitutively active RhoA-V14, indicating that in HeLa cells with decreased RhoA activity migration is inhibited by ionizing radiation, while RhoA overactivation enables cells to migrate after radiation treatment, in agreement with the results reported by Ridley in 2006 [24].

When compared with those displayed by parental HeLa cells, the proliferation and survival responses to *γ*-radiation of both HeLa cell lines expressing RhoA mutants are interesting, since they suggest that “fine-tuning” of RhoA activity impacts on DNA repair efficiency. Similar results were reported for canine T23 MDCK cells, where downregulation of RhoA activity by expression of RhoA-N19 decreased cell survival after toxin-mediated DNA double-strand break induction [2], showing that RhoA GTPase activity is important for survival after DNA damage in these cells. However, we observed that cells expressing the dominant negative RhoA-N19 and those expressing the constitutively active RhoA-V14 were equally susceptible to decreases in proliferation and survival, following *γ*-radiation. Interestingly, differential modulation of RhoA activity in HeLa cells led to population arrest at distinct stages of the cell cycle, which correlated with changes in the levels of cellular senescence and apoptosis observed in each cell line, following *γ*-radiation. These different “cell-fate” decisions seem to depend on the levels of RhoA activity, which in turn affect DNA damage sensing by the DDR pathway, directly reflecting in cell cycle phase-dependent triggering of cell proliferation inhibition followed by cell death. These data are in agreement with studies showing that DNA damage activates RhoA in an ATM-dependent manner and that RhoA activation is important for cell survival and proliferation, after treatment with low doses of *γ*-radiation [2, 25].

The effectiveness of radiotherapy treatment of human tumors is based (almost entirely) on the inability of cancer cells to repair radiation-induced DNA damage [2]. Given that the presence of DNA damage induces RhoA activation and triggers DNA repair mechanisms [2, 26], it is not surprising that DNA repair was more efficient in cells expressing the constitutively active RhoA-V14 mutant than in those expressing the dominant negative RhoA-N19, although DNA repair in HeLa-RhoA-V14 was still less efficient than that observed in parental HeLa cells. We detected increased levels of DNA damage relative to basal conditions for both mutant clones. We also observed increased levels of DNA damage and slow repair after inhibition of endogenous RhoA activity by C3 toxin expression, and RhoA inhibition drastically reduced the activity of the DNA repair pathways HR and NHEJ in HeLa cells. These data strongly suggest that RhoA GTPase is involved (possibly indirectly) in the regulation of DNA repair pathways, particularly in early repair. The similarities between our results and those obtained with HdCDT-induced DNA damage provide further support to our hypothesis that cytosolic RhoA signaling modulates nuclear genome integrity mechanisms [2, 27, 28].

Finally, our data on the effects of RhoA activity modulation on classical DNA damage response pathways suggest that RhoA is indirectly involved in the regulation of Chk1 and Chk2 activation after *γ*-radiation, because Chk1 (Ser345) and Chk2 (Thr-68) phosphorylation appeared attenuated in HeLa-RhoA-N19 cells and increased in HeLa-RhoA-V14, after exposure to *γ*-radiation.

**Figure 4:**
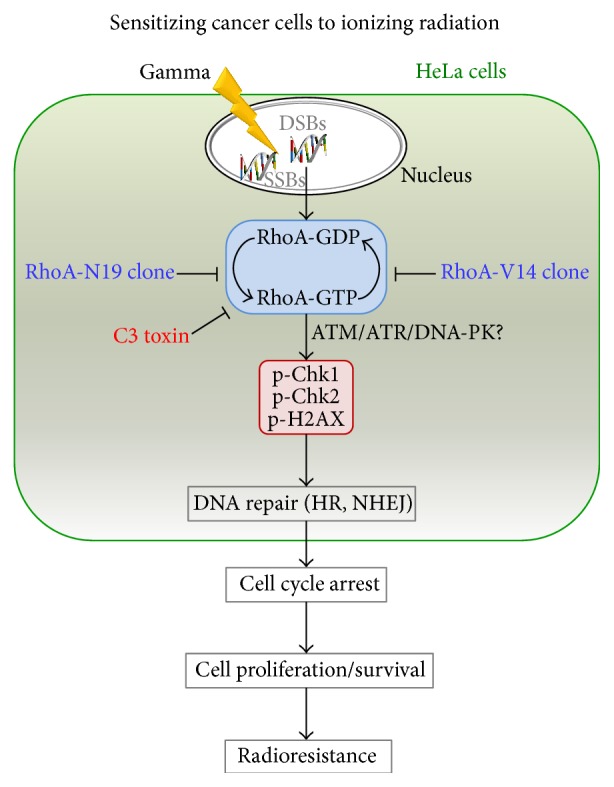
Model of the effects of RhoA activity modulation on global and specific DNA repair mechanisms, leading to increased cell proliferation or cell death, and reflecting in the radioresistance levels of cancer cells.

Chk1/Chk2 protein kinases are activated in response to DNA damage and are involved in DNA damage repair [29]. Pharmacological inhibition of Chk1/Chk2 induces cellular radiosensitivity, impairing DNA repair and triggering mitotic catastrophe, in the human colon cancer cell line HT-29 [30]. Thus, the attenuated phosphorylations of Chk1 and Chk2 in cells deficient in RhoA signaling may have impaired DNA repair (by HR and NHEJ) in these cells, which would explain the reductions in survival and proliferation, the specific cell cycle arrest pattern, and the increased levels of senescence and apoptosis observed in these cells. Overall, our data support the existence of a “cross talk” between RhoA signaling and DNA damage response and repair pathways in cancer cells (Figure 4), which may contribute to increased radioresistance. Importantly, these findings raise the interesting possibility that, in the clinic, the combination of chemotherapy using RhoA inhibitors followed by radiotherapy may lead to positive associations, for specific stages of cervical cancers.

## 5. Conclusions

Our findings provide strong evidence that positive or negative modulation of RhoA activity increases HeLa cell's sensitivity to *γ*-radiation treatment and therefore points to a possible clinical association of chemotherapy, using RhoA inhibitors, followed by radiotherapy sections for different stages of cervical cancers.

## Supplementary Material

Supplementary Figures S1 and S2 describe monolayer migration assays (by scratch assays) of HeLa cells and clones showing their motility under different stress conditions.
