# Supplementary material for: Modulation of RhoA GTPase Activity Sensitizes Human Cervix Carcinoma Cells to γ-Radiation by Attenuating DNA Repair Pathways
Source: Oxid Med Cell Longev. 2015 Nov 15;2016:6012642. doi: 10.1155/2016/6012642 (PMC4662998; doi:10.1155/2016/6012642)
Supplement: Supplementary file 1 — Supplementary Figures S1 and S2 describe monolayer migration assays (by scratch assays) of HeLa cells and clones showing their motility under different stress conditions. [file 6012642.f1.pdf]

Supplementary Figures

Supplementary Figure S1

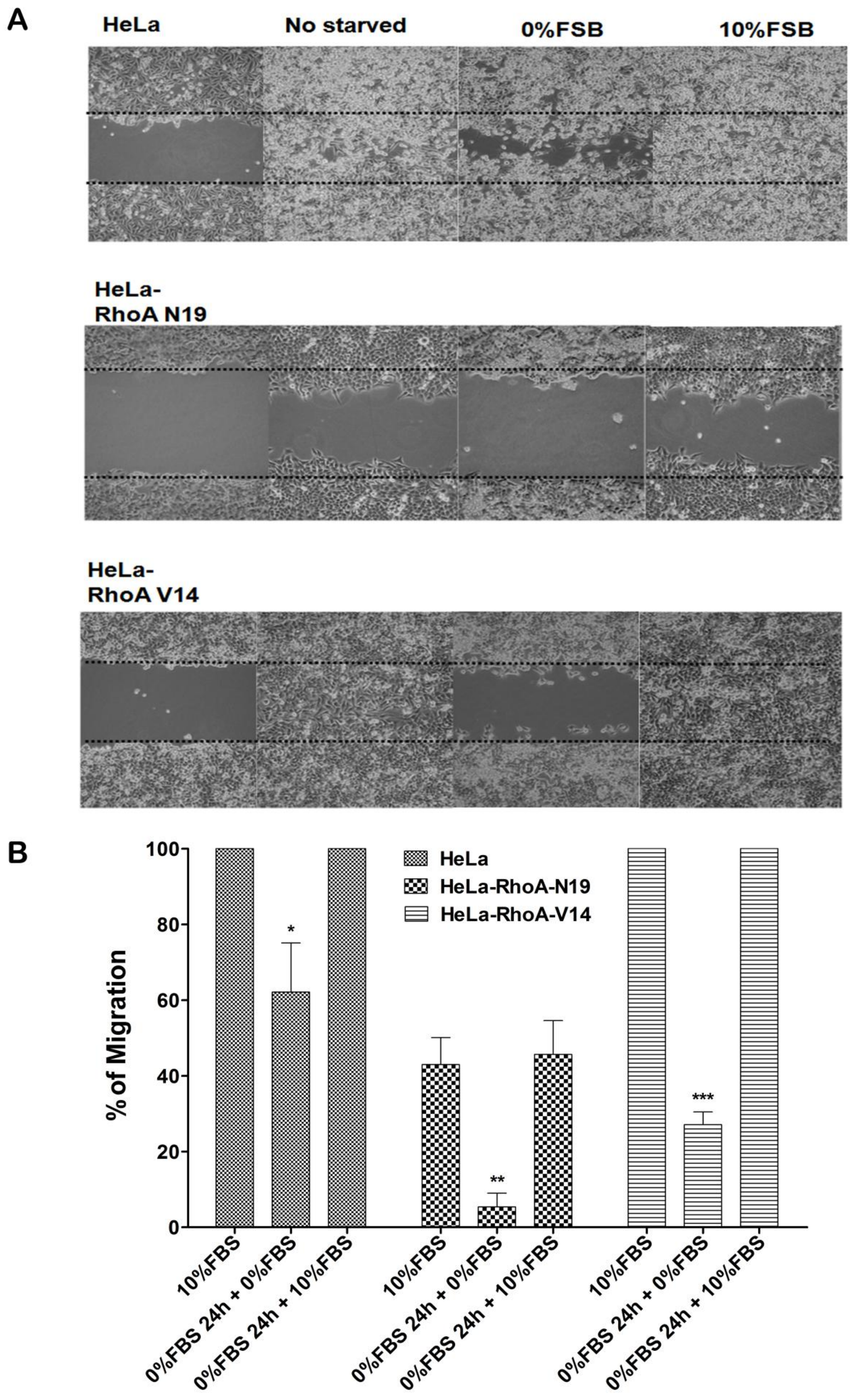

Supplementary Figure S1. Migration *in vitro* (by scratch assays) of HeLa cells expressing RhoA mutants. Confluent cell monolayers were scratched using micropipette tips, cells were allowed to migrate for 24h (at 37°C with 5% CO_2), and migration rates were calculated from light microscopy images of fixed cells, using the Cell-F software (Olympus). Cells were either grown and allowed to migrate in medium with 10% FBS (‘not starved’), or serum-starved (0% FSB) for 24h prior to scratch assays, and then allowed to migrate in medium with 0% or 10% FBS for additional 24h. A. Images of scratch assays using parental HeLa cells or HeLa-RhoA-V14 HeLa-RhoA-N19 clonal cell lines. B. Quantification of scratch assay data. Values represent mean ± SD from three independent experiments. *P<0.05, **P<0.001 and ***P<0.005 versus ‘not starved’ (by ANOVA).

Supplementary Figure S2

A

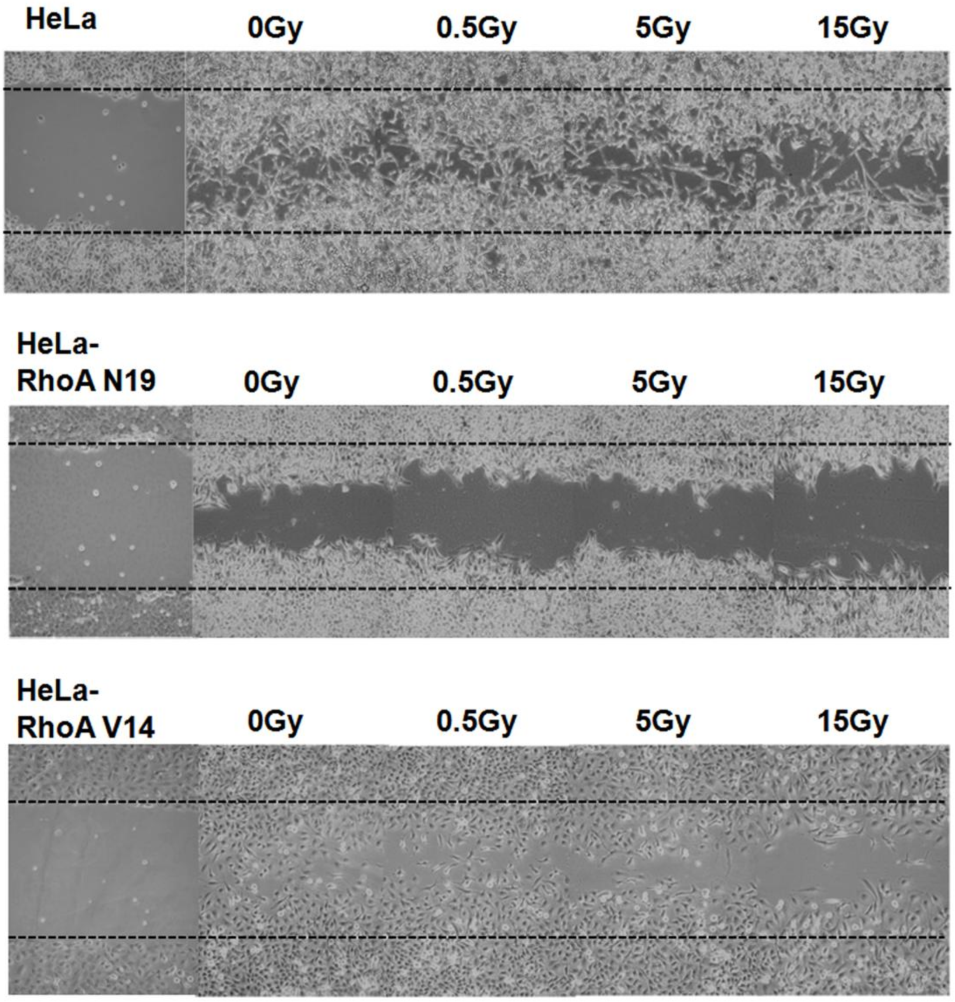

B

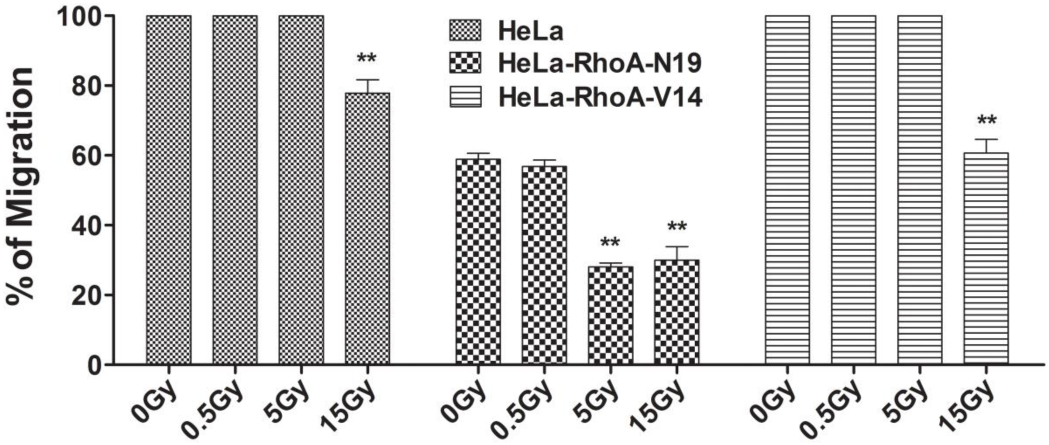

Supplementary Figure S2. Migration *in vitro* (by scratch assays) of HeLa cells expressing RhoA mutants, after γ-irradiation. Confluent cell monolayers were scratched using micropipette tips, cells were exposed to different doses of γ-radiation (0-15 Gy condition), and then allowed to migrate for by 24 h (at 37°C with 5% CO_2) in medium containing 10% FBS. Then, migration rates were calculated from light microscopy images of fixed cells, using Cell-F software (Olympus). A. Images of scratch assays of parental HeLa and HeLa-RhoA clonal cell lines. B. Quantification of scratch assay data. Values represent mean ± SD from three independent experiments. *P<0.05 and **P<0.001 relative to untreated (0 Gy) (by ANOVA).
